# Misspecification Strikes: ASTRAL can Mislead in the Presence of Hybridization, even for Nonanomalous Scenarios

**DOI:** 10.1093/molbev/msaf049

**Published:** 2025-03-07

**Authors:** Vu Dinh, Hector Baños

**Affiliations:** Department of Mathematical Sciences, University of Delaware, Newark, DE 197111, USA; Department of Mathematics, California State University, San Bernardino, CA 92407, USA

**Keywords:** ASTRAL, model misspecification, phylogenetic networks, network multispecies coalescent

## Abstract

ASTRAL is a powerful and widely used tool for species tree inference, known for its computational speed and robustness under incomplete lineage sorting. The method has often been used as an initial step in species network inference to provide a backbone tree structure upon which hybridization events are later added to such a tree via other methods. However, we show empirically and theoretically, that this methodology can yield flawed results. Specifically, we demonstrate that under the network multispecies coalescent model—including nonanomalous scenarios—ASTRAL can produce a tree that does not correspond to any topology displayed by the true underlying network. This finding highlights the need for caution when using ASTRAL-based inferences in suspected hybridization cases.

## Introduction

Inferring species networks remains a challenging problem in phylogenetics. An initial difficulty in determining species relationships, even without hybridization, is incomplete lineage sorting (ILS), which leads to gene tree discordance. The complexity of gene tree discordance increases when hybridization or other lateral gene transfer events are considered. The network multispecies coalescent model (NMSC) (Meng and Salter Kubatko 2009) is a standard probabilistic model for describing gene tree formation within species networks in the presence of ILS and hybridization. Although a variety of coalescent-based network inference methods exist, most are either restricted to a rather simple family of networks (known as level-1) (Solís-Lemus and Ané 2016; Allman et al. 2019; Kong et al. 2024; Allman et al. 2024), or lack scalability (Yu and Nakhleh 2015; Zhang et al. 2017; Wen and Nakhleh 2018).

A reasonable approach for inferring species networks, avoiding either oversimplification of the network or scalability issues, is first to infer a *“displayed”* (or *“backbone”*) tree, representing underlying tree-like relationships among species, and then inferring hybridization events on top of it using different techniques, notably, through Dsuite (Malinsky et al. 2021). Due to the absence of an established method for inferring a displayed tree, many researchers have used ASTRAL (Mirarab et al. 2014), a leading method for inferring species trees. This network inference methodology has been used in many works, for example (Owens et al. 2023; Zhou et al. 2022; Singh et al. 2022; Jensen et al. 2023; Sanderson et al. 2023; Ciezarek et al. 2024; Scherz et al. 2022; Yang et al. 2023; Lopes et al. 2023; Feng et al. 2022; Bernhardt et al. 2020; DeRaad et al. 2022; Herrig et al. 2024; Zhang et al. 2023; Zhou et al. 2023), among others. Such a pipeline relies on the assumption that ASTRAL accurately infers a tree displayed in the network, as adding hybridization edges to a tree not displayed in the network cannot yield the true network.

**Fig. 1.**
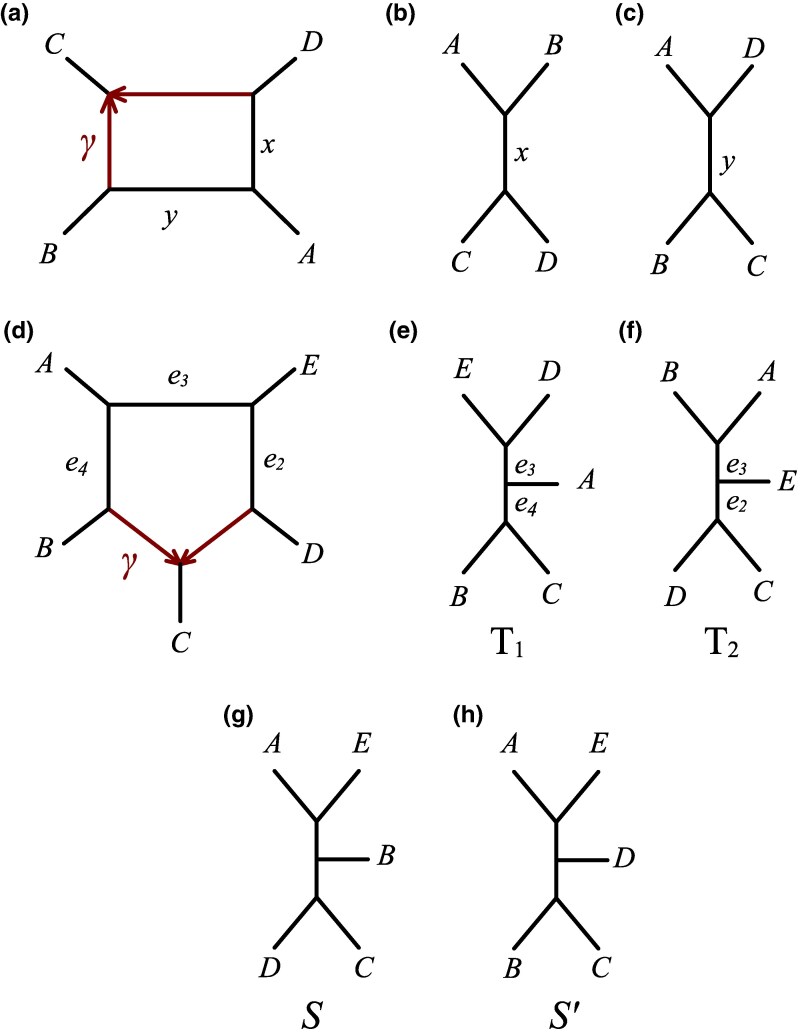
a) A semidirected network with a 4-cycle, hybrid parameter *γ*, and edge lengths *x* and *y* in coalescent units. b) The displayed tree obtained by removing the edge with probability *γ*. c) The displayed tree obtained by removing the edge with probability 1−γ. d) Similarly to above, a semidirected network *N* with a 5-cycle, hybrid parameter *γ*, and edge lengths $e_{2}$,$e_{3}$, $e_{4}$ in coalescent units. e) The displayed tree $T_{1}$ obtained from removing the edge with probability 1−γ. f) The displayed tree $T_{2}$ obtained from removing the edge with probability *γ*. g, h) The trees *S* and S′ from Theorem 1 and Corollary 2, respectively.

In this work, we demonstrate that under the NMSC, such a pipeline can be erroneous, as ASTRAL may fail to produce a reliable displayed tree. This failure results from model misspecification (given ASTRAL does not account for hybridization) and not from a flaw in ASTRAL’s approach to species tree inference. We demonstrate that even for relatively simple networks under the NMSC, such as one with a 5-cycle (see Fig. 1(d) for an example of such network), at least ∼6% of the parameter space can generate data that cause ASTRAL to infer a nondisplayed tree.

Challenges associated with using ASTRAL in the context of hybridization have been previously explored. Solís-Lemus et al. (2016) demonstrated that *anomalous networks* pose significant challenges for ASTRAL. Anomalous networks are such that a gene tree not displayed in the network occurs more frequently than one that is (Ané et al. 2024). Inferring anomalous networks presents challenges even for dedicated species network inference methods. Here, we show that ASTRAL’s behavior under the NMSC applies even to networks that are far from anomalous. In a different context, Long and Kubatko (2018) showed that ASTRAL can be misleading in the setting of a three-taxon isolation-with-migration model (with a similar structure to an anomalous network). However, their analysis incorporates continuous gene flow, which is not addressed by the standard NMSC. Lastly, in Pang and Zhang (2022), the authors primarily focus on cases where ASTRAL fails to recover the “major” tree, that is, the “predominant” tree structure within the network. However, the pipeline for network inference using ASTRAL, as described above, is not compromised in these instances, as the ASTRAL tree inferred may be displayed by the network. In such work, the authors describe these cases as “anomalous;” however, as mentioned before, here we adopt the term anomalous as defined in Ané et al. (2024).

## Background

### The NMSC

The NMSC is a probabilistic model describing the formation of gene trees, within a species network, in the presence of ILS and hybridization. In this model, gene lineages trace independently their ancestry backward in time through ancestral populations (the network edges). The probability that two lineages present in a population do not coalesce over time *t*, where *t* is measured in coalescent units, is given by $e^{-t}$. The coalescence rate depends on the number of lineages present, decreasing with each coalescent event. Lineages below a hybridization event trace their ancestry independently back to a particular population with probability *γ*, known as the hybridization parameter, which is specific to that population. This coalescent process continues indefinitely until all lineages have coalesced, resulting in the formation of a rooted gene tree.

Marginalizing over all taxa other than {A,B,C,D}, root location, and edge lengths, yields the probability that a gene tree displays each of the resolved topologies AB|CD,AC|  BD,AD|BC. Quartet gene tree probabilities are pivotal in species tree and network inference; for instance, ASTRAL’s statistical consistency relies on the property that, under the standard multispecies coalescent model (MSC), the most probable quartet gene tree shares the same topology as the quartet species tree (Allman et al. 2011).

As shown in Ané et al. (2024), for an arbitrary network, quartet gene tree probabilities are independent of root placement. Therefore, in this work, we generally depict networks without specifying the root, with only hybrid edges directed and nonhybrid edges left undirected, commonly known as *semidirected* networks.

For a network on *n* taxa with a single *n*-cycle—meaning a cycle with *n* edges when considered undirected—the distribution of gene trees under the NSMC model is a mixture of two gene tree distributions derived from the MSC (Rannala and Yang 2003). These distributions correspond to the displayed trees, which are obtained by removing one of the hybrid edges. See Fig. 1(a–f) for an example of a 4- and 5-cycle with their displayed trees.

As an illustrative example, and one that will be useful in a later section, the quartet probabilities for the 4-cycle network in Fig. 1(a) are given by:


(1) $$P(AB\;|\;CD)=(1-\gamma )\left(1-\frac{2}{3}e^{-x}\right)+\gamma \left(\frac{1}{3}e^{-y}\right),$$

(2) $$P(AD\;|\;BC)=(1-\gamma )\left(\frac{1}{3}e^{-x}\right)+\gamma \left(1-\frac{2}{3}e^{-y}\right),$$

(3) $$P(AC\;|\;BD)=(1-\gamma )\left(\frac{1}{3}e^{-x}\right)+\gamma \left(\frac{1}{3}e^{-y}\right).$$

In this particular case, P(AC|BD)<P(AB|CD),P(AD|BD), meaning that the two displayed trees are more frequent than the one not displayed by the network. Specifically,


(4) $$P(AB\;|\;CD)>P(AD\;|\;BC)\;\text{if and only if}\;(1-\gamma )(1-e^{-x})>\gamma \left(1-e^{-y}\right).$$

One of the key insights underlying our main results involves examining the differences in the probability between the most frequent quartet tree and its runner-up tree. For this scenario, this difference is given by


(5) $$P(AB\;|\;CD)-P(AD\;|\;BC)=(1-\gamma )(1-e^{-x})-\gamma \left(1-e^{-y}\right).$$

Particuarly, when γ=0, i.e. when there is no hybridization


$$P(AB\;|\;CD)-P(AD\;|\;BC)=1-e^{-x}.$$

### ASTRAL

ASTRAL is a quartet-based method for estimating species trees from multiple genes. The input of ASTRAL is a set of unrooted gene trees $T_{m}=\{T_{1},T_{2},\ldots ,T_{m}\}$, assumed to have arisen from the MSC on a tree. ASTRAL aims to find the (unrooted) species tree that agrees with the largest number of quartet trees induced by the set of gene trees. In principle, ASTRAL searches for a species tree T such that


$$\frac{1}{m}\sum_{q\in Q(T)}w_{m}(q,T_{m})$$

is maximized, where Q(T) is the set of quartet trees induced by T and $w_{m}(q,T_{m})$ is the number of the trees in $T_{m}$ that induce quartet topology *q*.

In ASTRAL, gene trees $T_{m}=\{T_{1},T_{2},\ldots ,T_{m}\}$ are assumed to be independently and identically distributed samples from the MSC on an unknown species tree T. Under this assumption, in the limit of large numbers of gene trees, the proportion of the trees in $T_{m}$ that induce a given quartet topology *q* converges to its quartet gene tree probabilities. Thus, if T′ is the true species tree, there is


$$\frac{1}{m}\sum_{q\in Q(T\prime )}w_{m}(q,T_{m})\approx \sum_{q\in Q(T\prime )}w(q,T\prime ),$$

where w(q,T′) is probability of observing the quartet topology *q* under the MSC model from a tree T′. The quantity of the right-hand side, which we will refer to as the expected ASTRAL score of the tree T′ and denote by A(T′), can be used as a proxy to study the behavior of ASTRAL on a large collection of error-free gene trees.

## ASTRAL Under Model Misspecification

In this section, we demonstrate theoretically and empirically that the ASTRAL tree inferred from data generated under the NMSC model for a 5-cycle network is not displayed by the network.

### The ASTRAL Tree for Perfect Data under the NMSC Model

Consider the 5 taxon network *N* in Fig. 1(d). The network *N* has a 5-cycle, hybrid parameter *γ*, internal nonhybrid edge lengths $e_{2}$,$e_{3}$, $e_{4}$ in coalescent units, displayed tree $T_{1}$ (Fig. 1(e)), and displayed tree $T_{2}$ (Fig. 1(f)). Note that, regardless of the placement of the root, each leaf is represented by only a single sample from the associated population. As a result, no coalescent events can occur along these edges, meaning their lengths do not affect the quartet gene tree topology probabilities. For this network, there are five distinct 4-taxon sets. Note that, as shown in Baños (2019), 5-cycle networks are not *quartet-anomalous*, meaning no quartet gene tree not displayed in the network occurs more frequently than one that is. We now show the main result of this work.

Theorem 1 Let *N* be the semidirected network on 5 taxa with displayed trees $T_{1}$ and $T_{2}$, as depicted in Fig. 1(d–f). For any branch length of *N*, let $y_{i}=\exp\;(-e_{i})$. If

(6) $$(1-y_{2})>\frac{\gamma }{1-\gamma }\left(1-y_{3}y_{4}\right)$$

and

(7) $$(1-y_{3})<\min\left(\frac{\gamma }{2-\gamma }\left(1-y_{4}\right),\frac{1-\gamma }{1+\gamma }\left(1-y_{2}\right)\right),$$

then for data generated from *N* under the NMSC, the tree S=(((A,E),B),C,D); (depicted in Fig. 1(g)), which is not displayed by *N*, has a higher expected ASTRAL score than both $T_{1}$ and $T_{2}$.

Proof. We begin by identifying, for each quartet network, the gene tree quartet with the highest and second-highest probability.

The subnetwork of *N* on A,B,C,D, is depiced in Fig. 1(a), where $x=e_{2}+e_{3}$ and $y=e_{4}$. Thus, the probabilities of the three gene tree quartets on this set of taxa are given by Equations (1)–(3). Note that, for $e_{2}$,$e_{3}$,$e_{4}>0$, then $\left(1-\gamma )(1-y_{2}y_{3})>(1-\gamma )(1-y_{2}\right)$ and $\gamma (1-y_{3}y_{4})>\gamma (1-y_{4})$. Together with Condition (6), this implies

$$(1-\gamma )(1-y_{2}y_{3})>\gamma \left(1-y_{4}\right).$$

Thus, by Equation (4), P(AB|CD)>P(AD|BC)  >P(AC|BD).

Using a similar reasoning, we observe that $\gamma (1-y_{4}y_{3})>\gamma (1-y_{3})$, which together with Condition (6), gives $\left(1-\gamma )(1-y_{2})>\gamma (1-y_{3}\right)$. Consequently, for the subnetwork on A,C,D,E, this yields P(AE|CD)>P(AC|DE)  >P(AD|CE).

Directly from Condition (6), we have $(1-\gamma )(1-y_{2})>$  $\gamma (1-y_{3}y_{4})$, which, analogously to the previous cases, gives P(BE|CD)>P(BC|DE)>P(BD|CE).

Following this approach, Condition (7) yields $\gamma (1-y_{4})>(1-\gamma )(1-y_{3}),$ implying P(AE|BC)>P(AB|CE)>P(AC|BE).

Finally, for the quartet on A,B,E,D, we note that the corresponding subnetwork is the tree AB|DE with internal branch $e_{3}$, giving gene tree probabilities P(AB|DE)>  P(AE|BD)=P(AE|BD).

From this, we observe that the expected ASTRAL score of any tree cannot exceed

$$\alpha^{*}=P(AB\;|\;CD)+P(AE\;|\;CD)+P(BE\;|\;CD)+P(AE\;|\;BC)+P(AB\;|\;DE).$$

The expected ASTRAL score for $T_{1}$, $T_{2}$, and S, which depends on their displayed quartets, is given by

$$\begin{array}{rl} A(T_{1}) & =P(AD\;|\;BC)+P(AC\;|\;DE)+P(BC\;|\;DE)\\ & \;+P(AE\;|\;BC)+P(AB\;|\;DE),\\ A(T_{2}) & =P(AB\;|\;CD)+P(AE\;|\;CD)+P(BE\;|\;CD)\\ & \;+P(AB\;|\;CE)+P(AB\;|\;DE),\\ A(S) & =P(AB\;|\;CD)+P(AE\;|\;CD)+P(BE\;|\;CD)\\ & \;+P(AE\;|\;BC)+P(AE\;|\;BD). \end{array}$$

The tree $T_{1}$ has several nonoptimal gene tree quartets. Particularly one on {A,C,D,E}. Thus, by Equation (5),

$$\begin{array}{r} \alpha^{*}-A(T_{1})\geq P(AE\;|\;CD)-P(AC\;|\;DE)\\ =(1-\gamma )(1-y_{2})-\gamma \left(1-y_{3}\right). \end{array}$$

The tree $T_{2}$ displays 4 of the optimal quartets, and only has a single nonoptimal topology, the one on {A,B,C,E}. Thus, by Equation (5),

$$\begin{array}{rl} \alpha^{*}-A(T_{2}) & =P(AE\;|\;BC)-P(AB\;|\;CE)\\ & =\gamma (1-y_{4})-(1-\gamma )\left(1-y_{3}\right). \end{array}$$

Note that S also displays 4 optimal quartets, and only gives a nonoptimal topology on {A,B,E,D}. Thus, by Equation (5),

$$\alpha^{*}-A(S)=P(AB\;|\;DE)-P(AE\;|\;BD)=1-y_{3}$$

By Condition (7), we have

$$1-y_{3}<\gamma (1-y_{4})-(1-\gamma )\left(1-y_{3}\right)\;\text{and}\;1-y_{3}<(1-\gamma )(1-y_{2})-\gamma \left(1-y_{3}\right).$$

Therefore, $A(S)>A(T_{1}),A(T_{2})$.

Analogously, by symmetry, the following corollary holds.

Corollary 2 Let *N* be the semidirected network as in Theorem 1. For any branch length of *N*, let $y_{i}=\exp\;(-e_{i})$. If

$$(1-y_{4})>\frac{1-\gamma }{\gamma }\left(1-y_{3}y_{2}\right)$$

and

$$(1-y_{3})<\min\left(\frac{\gamma }{2-\gamma }\left(1-y_{4}\right),\frac{1-\gamma }{1+\gamma }\left(1-y_{2}\right)\right)$$

then for data generated from *N* under the NMSC, then the tree S′=(((A,E),D),C,B); (depicted in Fig. 1(h)), which is not displayed in *N*, has a higher expected ASTRAL score than both $T_{1}$ and $T_{2}$.

While these theoretical results correspond to 5-taxon networks with a single 5-cycle, they can be extended to any arbitrary *n*-taxon network that has a 5-cycle as a subnetwork. Furthermore, in the Supplementary materials, we generalize Theorem 1 to *n*-cycle networks, with n≥5, demonstrating that these results also extend to networks containing a *k*-cycle as a subnetwork, where k≥5.

### Simulations

In the results above, the term data refers to a “perfect” sample under the NMSC. In this section, we present simulations that empirically validate our theoretical results. As a proof of concept, we first simulated a sample $T_{100K}$ of 100,000 gene trees from the rooted network N′, with extended Newick notation:


$$\begin{array}{rl} & ((((C\;:\;1)\#H1\;:\;1\;:\;\;:\;0.5,B\;:\;1)G\;:\;1.1,A\;:\;1)F\;:\;0.1,\\ & ((\#H1\;:\;1\;:\;\;:\;0.5,D\;:\;1)J\;:\;2,E\;:\;1)K\;:\;0.1)r; \end{array}$$

using PhyloCoalSimulations (Fogg et al. 2023) with default parameters. When unrooting N′, the resulting network is that in Fig. 1(d), where $e_{2}=2$, $e_{3}=0.2$, $e_{4}=1.1$, γ=0.5, with all other edges set to 1, thus satisfying the conditions of Theorem 1.

Using default parameters, we then ran ASTRAL-III (Zhang et al. 2018) v.5.7.8 on $T_{100K}$. As expected, the resulting ASTRAL tree is S. We primarily used ASTRAL-III in this work, as it has been the most widely adopted tool for the network inference pipeline described in the introduction. However, for completeness, we also input $T_{100K}$ through ASTRAL-IV v1.19.4.6 (Zhang and Mirarab 2022), and ASTRAL-PRO v1.19.3.6 (Zhang et al. 2020), with default parameters. For all methods, the tree recovered is S. In $T_{100K}$, the two most frequent unrooted gene tree topologies agree with the displayed trees $T_{1}$ and $T_{2}$ with frequencies ∼0.174 and ∼0.214, respectively. The third and fourth most frequent topologies are (((B,E),A),C,D); and S, with frequencies ∼0.131 and ∼0.130, respectively, indicating that N′ is not anomalous (nor quartet-anomalous as mentioned before).

To estimate the size of the parameter space of a 5-cycle network *N* whose data would yield an ASTRAL tree not displayed by *N*, we uniformly sampled $10^{6}$ points from $[0,1{]}^{4}$. The first three entries correspond to transformed branch lengths $\log\;(e_{2}),\log\;(e_{3}),\log\;(e_{4})$, corresponding to sampling edge lengths from an exponential distribution with mean 1, and the remaining one to the hybridization parameter *γ*. We denote by *Θ* the set of parameters that satisfy either condition of Theorem 1 or Corollary 2. We found that ∼0.06 of the samples are in *Θ*.

**Fig. 2.**
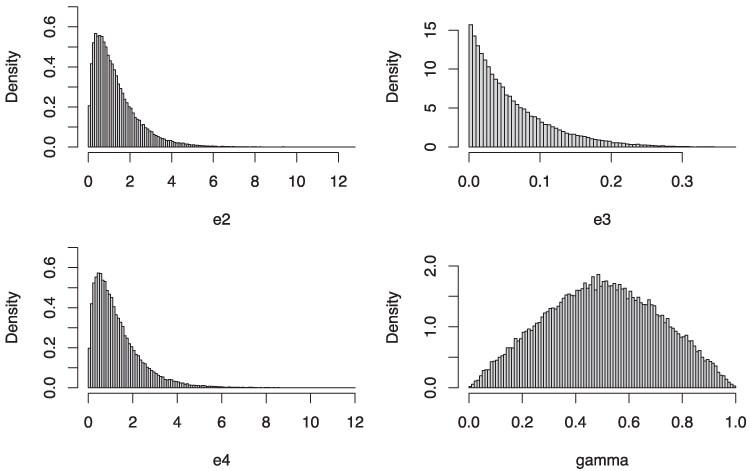
Histograms illustrating the range of parameters in *Θ*, i.e. those satisfying the hypothesis of Theorem 1 or Corollary 2. In each histogram, the *y*-axis represents the density, while the *x*-axis corresponds to a specific parameter. The histograms are organized from left to right and top to bottom, corresponding to $e_{2}$ (with mean value ∼1.3), $e_{3}$ (mean ∼0.06), $e_{4}$ (mean ∼1.3), and *γ* (mean ∼0.5). Note that all edge lengths ($e_{2},$  $e_{3}$, and $e_{4}$) are expressed in coalescent units.

Figure 2 illustrates the wide range of parameters in *Θ*. Each histogram in the figure represents the distribution of a specific parameter, derived from the points in *Θ* from the $10^{6}$ sampled parameters. To demonstrate that these parameters are indeed empirically problematic for ASTRAL, we simulated 1,000 gene trees for 100 randomly sampled parameter sets in *Θ*. We then ran ASTRAL-III with default parameters and found that in 86 out of the 100 trials, the ASTRAL tree was not displayed in the network. In the 14 cases where the tree was displayed, we simulated 50,000 gene trees. In all 14 instances, ASTRAL failed to recover a displayed tree in the larger data sets, suggesting the initial successes were likely due to finite sample error.

Note that the average values for each parameter (as shown in the figure captions) represent biologically reasonable estimates. For edge lengths, there are some cases where the parameters may be unrealistic, for example, edge lengths greater than 5 coalescent units. Nonetheless, the majority of edge lengths fall below this threshold. For the edge $e_{3}$, there are many instances where the edge length is very short, this may be the result of rapid divergences or populations with large effective sizes. Regarding the hybridization parameter, the results suggest that most issues arise from events with a strong hybridization signal (*γ* close to 0.5). However, even in cases with minimal hybridization (e.g. when *γ* is close to 0 or 1), erroneous inference can still occur.

To further investigate the parameter space, we restricted *γ* to different hybridization levels. Table 1 displays the proportion of parameters in *Θ* across different *γ* ranges. As *γ* approaches 0.5 (indicative of a strong hybridization signal) more edge length sets are satisfying the conditions of our results. For example, Table 1 shows that when *γ* is between 0.4 and 0.6, approximately, 10% of the parameter space is in *Θ*. Finally, we showed that even when *γ* values are close to 0 or 1, indicating a low hybridization signal, parameters still exist that can lead to erroneous inferences by ASTRAL (last column of Table 1).

**Table 1.** The proportion of parameters in *Θ* (i.e. satisfying the conditions of Theorem 1 or Corollary 2).

Range for *γ*	(0,1)	(0.2,0.8)	(0.4,0.6)	(0,0.1)∪(0.9,1)
Proportion of parameters in *Θ*	0.06	0.08	0.10	0.01

Each proportion is calculated from values of *γ* within *Θ*, based on $10^{6}$ parameter samples. In each sample, *γ* is uniformly selected within the specified interval, while $e_{2}$, $e_{3}$, and $e_{4}$ are sample edges from an exponential distribution with mean 1.

## Discussion

This study highlights the downsides of species network inference using a pipeline in which a tree is first inferred with ASTRAL. Our results indicate that for data generated under the NMSC, the inferred ASTRAL tree can differ from the one displayed in the network. We emphasize that the issues with ASTRAL arise from model misspecification rather than limitations inherent to ASTRAL’s theoretical framework. Nonetheless, our results show the need for a tool that can reliably infer a displayed tree from a network. In principle, it is possible to quantify the fit between data and a fixed network, for example, using tools such as those developed by Cai and Ané (2020). However, no existing method allows testing, whether a specific tree is displayed in a network. If there were a reliable method for inferring a displayed tree, the pipeline discussed would not, in principle, present fundamental issues. A methodology to infer a displayed tree was introduced in Pyron et al. (2024), where the authors proposed a heuristic approach to infer displayed trees. While promising, this methodology is not easily scalable and has theoretical limitations, highlighting a need for further development.

While here we showed that the ASTRAL tree can differ from a displayed tree by a single nearest neighbor interchange (NNI) move, additional simulations in the Supplementary material, show that this discrepancy can become more pronounced in networks with larger cycles as well as with more hybridization events. We also anticipate similar issues occur in other quartet-based methods under model misspecification, impacting not only tree inference but also network inference methods that assume a fixed network level, particularly those using a pseudolikelihood approach (Solís-Lemus and Ané 2016; Kong et al. 2024). Addressing these limitations requires an alternative framework, which we will explore in future work.

## Supplementary Material

msaf049_Supplementary_Data

## Data Availability

The data underlying this article were entirely simulated and can be easily replicated using the methods described in the manuscript.
