# Supplementary material for: Misspecification Strikes: ASTRAL can Mislead in the Presence of Hybridization, even for Nonanomalous Scenarios
Source: Mol Biol Evol. 2025 Mar 7;42(3):msaf049. doi: 10.1093/molbev/msaf049 (PMC11934270; doi:10.1093/molbev/msaf049)
Supplement: msaf049_Supplementary_Data [file msaf049_supplementary_data.pdf]

Supplementary material: "Misspecification Strikes: ASTRAL can Mislead in the Presence of Hybridization, even for Non-Anomalous Scenarios"

Vu Dinh[1,*] and Hector Banos[2]

[1] *Department of Mathematical Sciences, University of Delaware*
[2] *Department of Mathematics, California State University, San Bernardino*
**Email: vucdinh@udel.edu*

ASTRAL under model misspecification: a n-cycle case

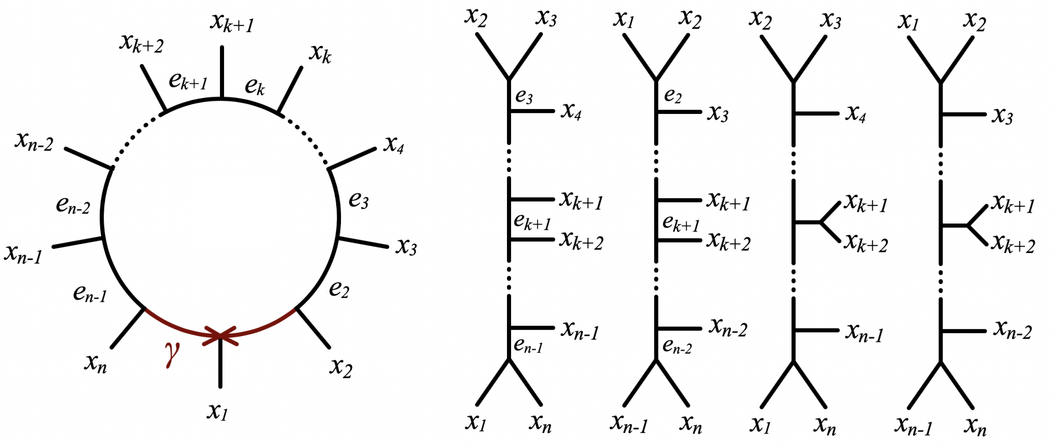

Figure 1: In order of appearance from left to right: 1) an n-cycle network N with hybrid taxon x_1, hybrid parameters γ and $1-\gamma$, edge lengths in coalescent units e_i; 2) The displayed tree $\mathbb{T}_1$ obtained after removing the edge with hybrid parameter $1-\gamma$; 3) The displayed tree $\mathbb{T}_2$ obtained after removing the edge with hybrid parameter γ; 4) The tree $\mathcal{S}_1$ obtained from $\mathbb{T}_1$ after a single NNI move of the leave x_{k+1}; 5) The tree $\mathcal{S}_2$ obtained from $\mathbb{T}_2$ after a single NNI move of the leave x_{k+1}.

For an arbitrary n, we consider the two trees $\mathbb{T}_1$ and $\mathbb{T}_2$ displayed by the n-cycle network, as appeared in Figure 1. For $1 < i < n$, the length of the inner edge connecting x_i and x_{i+1} are consistent between the two displayed trees $\mathbb{T}_1$, $\mathbb{T}_2$ and are denoted by e_i.

Theorem. *Let N be a semi-directed network on n taxa with displayed trees $\mathbb{T}_1$ an $\mathbb{T}_2$, as depicted in Figure 1. For any branch length e_i, let $y_i = \exp(-e_i)$. Given $1 < k < n-2$, let $\mathcal{S}_1, \mathcal{S}_2$ be the trees obtained from $\mathbb{T}_1, \mathbb{T}_2$ by a single NNI move that attaches x_{k+1} to the pendent edge of x_{k+2} in $\mathbb{T}_1, \mathbb{T}_2$, respectively (as depicted in Fig. 1). If the length of the edges of the network satisfy*

$$1 - y_{k+1} < \min\left\{\frac{\gamma}{k-\gamma}(1-y_{k+2}), \frac{1-\gamma}{n-k-2+\gamma}(1-y_k)\right\} \qquad (*)$$

then either $\mathcal{S}_1$ or $\mathcal{S}_2$ has higher expected ASTRAL score than both $\mathbb{T}_1$ and $\mathbb{T}_2$.

Proof. Similar to the case of 5 taxa (detailed in the main manuscript), we first identify the optimal topologies (i.e., the quartet topology with the highest probability) on all 4-taxon sets of the network. For all 4-taxon sets of the network, we use two criteria

(Q1) Whether x_1 is a member of the set

(Q2) Whether both x_{k+1} and x_{k+2} are members of the set

to classify them into different categories using Table 1.

Type	Q1	Q2	Quartet Set
1	No	No	$\{x_a, x_b, x_c, x_d\}$
2	No	Yes	$\{x_{k+1}, x_{k+2}, x_a, x_b\}$
3	No	Yes	$\{x_a, x_b, x_{k+1}, x_{k+2}\}$
4	No	Yes	$\{x_a, x_{k+1}, x_{k+2}, x_b\}$
5	Yes	No	$\{x_1, x_a, x_b, x_c\}$
6	Yes	Yes	$\{x_1, x_{k+1}, x_{k+2}, x_c\}$
7	Yes	Yes	$\{x_1, x_a, x_{k+1}, x_{k+2}\}$

Table 1: Table showing the 7 different classifications of quartets based on Criteria Q1 and Q2 and accounting for the order of the taxa in the network. We assume $1 < a < b < c < d$. Note also that for any set in the 'Quartet Set' column, its elements are ordered by increasing subscript, for example for Type 2, $k+1 < k+2 < a < b$, while for Type 4 $a < k+1 < k+2 < b$.

We have the following remarks.

- For taxon sets of Types 1, 2, and 3: The topologies displayed by $\mathbb{T}_1$, $\mathbb{T}_2$, $\mathcal{S}_1$, $\mathcal{S}_2$ are the same.
- For taxon sets of Type 4:
  - $\mathbb{T}_1$, $\mathbb{T}_2$ (both) display the quartet topology $x_a x_{k+1} | x_{k+2} x_b$ with inner edge length e_{k+1}
  - $\mathcal{S}_1$, $\mathcal{S}_2$ (both) display the quartet topology $x_a x_b | x_{k+1} x_{k+2}$
- For taxon sets of Type 5:
  - $\mathbb{T}_1$, $\mathcal{S}_1$ (both) display the quartet topology $x_1 x_c | x_a x_b$
  - $\mathbb{T}_2$, $\mathcal{S}_2$ (both) display the quartet topology $x_1 x_a | x_b x_c$
- For taxon sets of Type 6:
  - $\mathbb{T}_1$, $\mathcal{S}_1$, $\mathcal{S}_2$ display the quartet topology $x_1 x_c | x_{k+1} x_{k+2}$ with inner edge length $\sum_{i=k+2}^{c-1} e_i$ on $\mathbb{T}_1$
  - $\mathbb{T}_2$ displays the quartet topology $x_1 x_{k+1} | x_{k+2} x_c$ with inner edge length e_{k+1}
- For taxon sets of Type 7:
  - $\mathbb{T}_2$, $\mathcal{S}_1$, $\mathcal{S}_2$ display the quartet topology $x_1 x_a | x_{k+1} x_{k+2}$ with inner edge length $\sum_{i=a}^{k} e_i$ on $\mathbb{T}_2$
  - $\mathbb{T}_1$ displays the quartet topology $x_1 x_{k+2} | x_a x_{k+1}$ with inner edge length e_{k+1}.

We note that Condition (*) implies that

$$\gamma(1 - y_{k+2}) > (1-\gamma)\,(1 - y_{k+1}) \quad \text{and} \quad (1-\gamma)(1-y_k) > \gamma\,(1 - y_{k+1})\,.$$

Thus, $\mathcal{S}_1$ and $\mathcal{S}_2$ are optimal on all four-taxon sets of Type 6 and Type 7.

For convenience, for a given four-taxon set, we use the term "regret" of a quartet on that taxon set to refer to the (absolute) difference between the probability of a quartet to that of the optimal topology. In other words, the regret of a quartet is non-negative and is zero if the quartet is optimal.

We note that across all four-taxon sets of Type 5, $\mathbb{T}_1$ and $\mathcal{S}_1$ have the same topologies, and thus the same regrets). Similarly, $\mathbb{T}_2$ and $\mathcal{S}_2$ have the same regrets on all four-taxon sets of this type. Let Δ be the difference in total regret across all four-taxon sets of Type 5 between $\mathbb{T}_1$ and $\mathbb{T}_2$, we will choose a tree $\mathcal{S}$ as follows: If Δ is positive, we choose $\mathcal{S} = \mathcal{S}_2$; otherwise, $\mathcal{S} = \mathcal{S}_1$. With this choice of $\mathcal{S}$, the total regret across all four-taxon sets of Type 5 of $\mathcal{S}$ do not exceed those of $\mathbb{T}_1$ and $\mathbb{T}_2$.

We can bound the regrets across all 4-taxon sets that are not of Type 5 for the trees $\mathcal{S}$, $\mathbb{T}_1$, and $\mathbb{T}_2$ as follows.

1. $\mathcal{S}$ only has sub-optimal topology on taxon sets of Type 4, and there are $(k-1)(n-k-2)$ such four-taxon sets. Thus, the total regret of $\mathcal{S}$ on all 4-taxon sets that are not of Type 5 is
$$(k-1)(n-k-2)\left(1-y_{k+1}\right).$$

2. $\mathbb{T}_2$ has sub-optimal topology on all four-taxon sets of Type 6, and there are $n-(k+2)$ such four-taxon sets. The total regret of $\mathbb{T}_2$ on all four-taxon sets that are not of Type 5 is bounded from below by
$$(n-k-2)\left(\gamma(1-y_{k+2})-(1-\gamma)\left(1-y_{k+1}\right)\right).$$

3. $\mathbb{T}_1$ has sub-optimal topology on all four-taxon sets of Type 7, and there are k such four-taxon sets. The total regret of $\mathbb{T}_1$ on all 4-taxon sets that are not of Type 5 is bounded from below by
$$(k-1)\left((1-\gamma)(1-y_k)-\gamma\left(1-y_{k+1}\right)\right).$$

We conclude that if
$$1-y_{k+1} < \min\left\{\frac{\gamma}{k-\gamma}\left(1-y_{k+2}\right), \frac{1-\gamma}{n-k-2+\gamma}\left(1-y_k\right)\right\}$$
then the tree $\mathcal{S}$ has higher expected ASTRAL score than both $\mathbb{T}_1$ and $\mathbb{T}_2$.

□

Simulation: 8-cycle network

Similarly to the main text, we simulated a dataset $\mathcal{T}'_{100K}$ of 100,000 gene trees from the 8-cycle network N'' (depicted in Figure 2 along with its displayed trees). The network is represented in extended Newick notation as:

```
"((((((E:1)H1:0.5::0.5,D:1)L:0.6,C:1)K:0.6,B:1)J:.9,A:1)I:0.01,
    (((H1:0.5::0.5,F:1)O:1.5,G:1)P:1.5,H:1)Q:0.01)r;"
```

Using default parameters, we ran ASTRAL-III v.5.7.8 on $\mathcal{T}'_{100K}$. The resulting ASTRAL tree is:

```
"(D,(C,(B,((A,H),(E,(F,G))))));"
```

This tree, shown in Figure 2, is more than one NNI move away from either of the displayed trees.

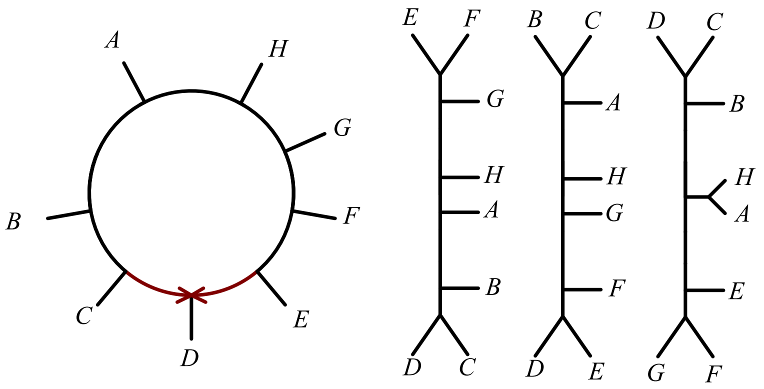

Figure 2: In order of appearance from left to right: 1) an 8-cycle network N''; 2) One of the displayed trees of N''; 3) The other displayed tree of N''; 4) The tree obtained from ASTRAL-III on the sample $\mathcal{T}'_{100K}$.

Simulation: Level-2 network

Similarly to the case above, we simulated a dataset $\mathcal{T}^*_{100K}$ of 100,000 gene trees from the network N^* depicted in Figure 3 (where its displayed trees are also depicted in such a Figure). The network is represented in extended Newick notation as:

```
"((((((E:1)H1:0.5::0.5,D:1)LL:0.6,C:1)KK:0.6,B:1)JJ:0.9,A:1)II:0.01,
(H:1,(((I:1,(J:1,(K:1)H2:0.5::0.5)Y:1.2)X:0.0001,
((H1:0.5::0.5,F:1)O:1.5,G:1)P:1)V:0.001,
(M:1,(H2:0.5::0.5,L:1)AA:1)CC:0.001)W:0.5)Q:0.01)r;"
```

Using default parameters, we ran ASTRAL-III v.5.7.8 on $\mathcal{T}^*_{100K}$. The resulting ASTRAL tree is:

```
"(L,((J,K),(M,(I,((G,F),((H,A),(E,(B,(C,D)))))))));"
```

Similarly to the case above, this tree, shown in Figure 3, is more than one NNI move away from either of the displayed trees.

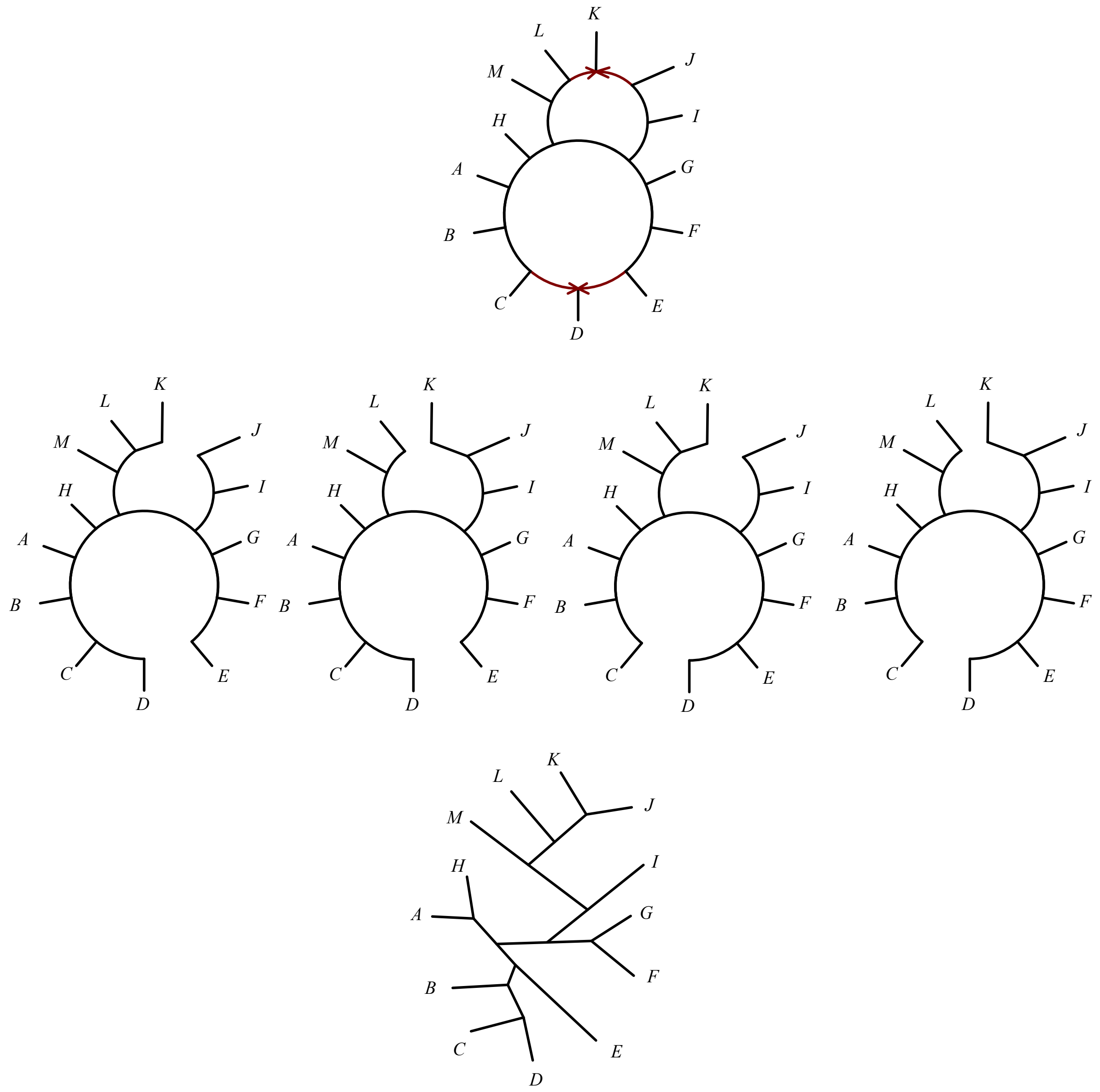

Figure 3: (Top) The network N^* with two hybridization events; (Center) The four displayed trees of N^*; (Bottom) The tree obtained from ASTRAL-III on the sample $\mathcal{T}^*_{100K}$.
